# Supplementary material for: Instantaneous antidepressant effect of lateral habenula deep brain stimulation in rats studied with functional MRI
Source: eLife. 2023 Jun 1;12:e84693. doi: 10.7554/eLife.84693 (PMC10234627; doi:10.7554/eLife.84693)
Supplement: Figure 4—source data 1. — The Pearson’s correlation coefficient r between beta values of blood-oxygenation-level-dependent (BOLD) responses and performance indicators of open field test (OFT) across rats was calculated for the cingulate cortex (Cg), retrosplenial cortex (RS), medial prefrontal cortex (mPFC), lateral habenula (LHb), and interpeduncular nucleus (IPN)/ventral tegmental area (VTA), including change of average speed, defined as the ratio of average speed with deep brain stimulation (DBS) and without DBS (non-DBS), number of entries into the center and distance traveled in center. [file elife-84693-fig4-data1.docx]

|  | | **Beta value** | | | | |
| --- | --- | --- | --- | --- | --- | --- |
|  |  | **Cg** | **RS** | **mPFC** | **LHb** | **IPN / VTA** |
| **Change of average speed (DBS/non-DBS)** | **Pearson correlation coefficient (*r*)** | **6.5 e^-4^** | **0.30** | **0.34** | **-0.04** |  |
|  | ***p*** | **0.99** | **0.21** | **0.15** | **0.87** |  |
| **Number of entries into center** | **Pearson correlation coefficient (*r*)** | **-0.18** | **0.28** | **0.18** | **-0.24** | **0.11** |
|  | ***p*** | **0.45** | **0.25** | **0.46** | **0.33** | **0.66** |
| **Distance traveled in the center (cm)** | **Pearson correlation coefficient (*r*)** | **-0.09** | **0.35** | **0.17** | **-0.22** | **0.11** |
|  | ***p*** | **0.72** | **0.15** | **0.49** | **0.36** | **0.65** |
